# Circadian changes of autonomic function in patients with zoster‐associated pain: A heart rate variability analysis

**DOI:** 10.1002/brb3.3489

**Published:** 2024-04-30

**Authors:** Peng Mao, Hui‐Min Hu, Ran Li, Yuan‐Jing Zhang, Yi Zhang, Yi‐Fan Li, Bi‐Fa Fan

**Affiliations:** ^1^ Graduate School of Beijing University of Chinese Medicine Beijing China; ^2^ Department of Pain Medicine China‐Japan Friendship Hospital Beijing China

**Keywords:** autonomic function, circadian changes, heart rate variability, zoster‐associated pain

## Abstract

**Objective:**

To investigate the circadian changes of the autonomic function in patients with zoster‐associated pain (ZAP).

**Methods:**

A total of 37 patients with ZAP from April 2022 to October 2022 were enrolled as the observation group, and 37 normal volunteers at the same time were selected as the control group. All participants were required to wear a 24‐h Holter, which was used to compare the heart rate variability (HRV) between the two groups. HRV analysis involved time‐ and frequency‐domain parameters.

**Results:**

There was no statistically significant difference in general information between two groups. Patients with ZAP had an increased mean heart rate and decreased the standard deviation of normal‐to‐normal (SDNN) R–R interval, the root mean square of the differences (RMSSD) in successive RR interval, low frequency (LF), and high frequency (HF) compared with control groups in all periods (*p* < .05). The ratio of LF/HF between two groups had no significant difference (*p* = .245). SDNN had no significant difference between day and night in the control group (*p* > .05), whereas SDNN of ZAP patients in night period was reduced than that in day period (*p* < .001). The level of RMSSD during the day was lower than those at night in the control group (*p* < .05), whereas no significant difference of RMSSD between two periods was observed in patients with ZAP (*p* > .05).

**Conclusion:**

The results of this study indicated that ZAP contributes to the decline of autonomic nervous system (ANS) function, especially parasympathetic components. The patients with ZAP lost parasympathetic advantage and had a worse ANS during the night.

## Abstract 2

In this work, we conducted an observational study of Circadian Changes of Autonomic Function in Patients with Zoster‐associated Pain via HRV. To our best knowledge, our study is one of the rare and comprehensive attempts to explore circadian changes of autonomic function in patients with ZAP via 24‐hour heart rate variability analysis. The results of this study indicated that ZAP contributes to change in the ANS resulting in decreased the function of ANS and PNS in all periods. The patients with ZAP lost parasympathetic advantage and had a worse ANS during the night.

## Graphical abstract

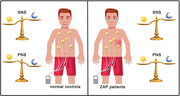

## 1 INTRODUCTION

Herpes zoster (HZ) is a neurocutaneous viral disease that occurs in individuals infected by varicella‐zoster virus (VZV) (Patil et al., 2022). Zoster‐associated pain (ZAP), a typical and intractable neuropathic pain and the most common complication of HZ, results from the activation of the latent VZV in the dorsal root ganglions (DRGs) in patients with low cell‐mediated immunity (Depledge et al., 2018; Kaikai & Dowling Evans, 2022). Reactivation and continuous replication of VZV contribute to an acute neuroinflammatory response accompanied by hemorrhagic necrosis and demyelinating changes of nerve cells, causing neuropathic pain (Hadley et al., 2016). Both severe acute HZ and postherpetic neuralgia (PHN) give rise to psychosocial dysfunction, consisting of anxiety, depression, sleep disturbance, cognitive decline, chronic fatigue, loss of appetite, and so on (Lee et al., 2016; Pickering & Leplege, 2011). ZAP can result in significant suffering, economic loss, and medical burden (Pan et al., 2022).

Autonomic dysfunction has long been thought to be associated with the pathogenesis of a variety of chronic pain diseases. In physiological status, there is no functional connection between sympathetic postganglionic neurons and peripheral afferent sensory neurons, and the excitation of sympathetic neurons does not result in the sensitivity of primary sensory neurons. Once nerve is injured, coupling phenomenon happens between the sympathetic postganglionic fibers and the peripheral nerve afferent fibers, which contributes to peripheral sensitization in turn (Pertin et al., 2007). What's more, parasympathetic nerve function is also involved in pain sensitization. Decreasing vagal activity leads to greater somatic and visceral input via the spinothalamic track, which renders a mechanism for decreased pain threshold and increased pain sensitivity in those with chronic pain (Koenig, Falvay et al., 2016).

Heart rate variability (HRV) refers to the characteristic beat‐to‐beat variability in the heart rate time series, generated by the regulation of the sinus node by the ANS(Burr et al., 2006). In healthy individual, sympathetic and vagus nerves are in a dynamic balance. However, HRV mostly decreases when a disorder disrupts autonomic homeostasis (Ishaque et al., 2021). Therefore, HRV can be used to assess the condition and interactions of the sympathetic nervous system (SNS) and PNS.

To our knowledge, no previous study used 24‐h HRV analysis to evaluate autonomic function in patients with ZAP. Given this knowledge gap, this study aims to assess the autonomic function and circadian rhythm of ZAP patients via HRV.

## 2 METHODS

### 2.1 Study design and participants

This observational study was conducted with patients diagnosed with ZAP and enrolled from China from April 2022 to October 2022. Diagnosis of ZAP was confirmed by a pain physician. Patients who met the diagnosis were enrolled in the study and arranged to complete blood routine, biochemical examinations, and an electrocardiogram to confirm whether there were other factors interfering with HRV.

The inclusion criteria for the patients are as follows: (1) aged >18 years; (2) fulfill features of the pathogenesis of ZAP strictly: previous infection with shingles, pain distribution consistent with neuroanatomical features, and paresthesia at the lesion site; and (3) visual analog scale (VAS) ≥4.

Participants who met one of the following were excluded: (1) severe comorbidities such as cardiopathy, cerebrovascular disease, intracranial space‐occupying lesions, and/or gastrointestinal, renal, hepatic, hematologic, respiratory, or endocrine disease, or psychosis; (2) local skin allergies or other serious skin diseases; and (3) other neurological injury diseases or painful disorders.

Subjects in the control group were recruited from healthy people. As same as ZAP patients, laboratory tests for healthy people were finished to confirm their health. The study was approved by the ethics committee of the hospital (2022‐KY‐146). All subjects provided written informed consent prior to participation.

### 2.2 Pain evaluation

The degrees of pain in patients with ZAP were evaluated through VAS. VAS is a 100‐mm continuous horizontal line with the 0‐mm end meaning no pain and the 100‐mm end representing the most severe pain; the in‐between part means different degrees of pain (Williamson & Hoggart, 2005).

### 2.3 HRV analysis

The data about HRV was obtained from 24‐h Holter (DMS300‐4A, DMS) and analyzed by DMS Holter software V12. All the participants were asked to maintain a normal lifestyle while wearing the device, avoid contact with strong electromagnetic interference and other examinations, and avoid the electrode sheet falling off. Every subject was demanded to refrain from undertaking exercise, drinking tea or coffee, or smoking for 12 h prior to the HRV measurement. In addition, every patient was asked to temporarily stop the use of painkillers and antiepileptic drugs.

HRV analysis is broadly divided into linear and nonlinear methods. Among the linear methods, time‐domain‐ and frequency‐domain analysis were performed in this study. Time‐domain parameters are used for the assessment of the ability of the ANS to control body, including the standard deviation of normal‐to‐normal (SDNN) and the root mean square of the differences (RMSSD). SDNN reflects the overall ANS function. RMSSD responds to vagal tone (Hirfanoglu et al., 2018; Tiwari et al., 2021).

Frequency‐domain parameters, as a power spectral analysis, signify the occurrence of oscillations in the cardiac rhythm in certain frequencies and provide the information to quantitatively evaluate ANS function. High frequency (HF), ranging between 0.15 and 0.40 Hz, is relevant to parasympathetic activity. Low frequency (LF), ranging between 0.04 and 0.15 Hz, indicates sympathetic activity and some vagal influences. LF/HF, as an index of the global sympathy‐vagal balance, is calculated to exclude parasympathetic components in the LF. The higher the ratio, the stronger the SNS function (Hirfanoglu et al., 2018; Jang & Seol, 2021; Tiwari et al., 2021).

HRV parameters are evaluated by dividing them into two different time periods (daytime and nighttime). The day period was taken as the time between 06:00 and 21:59, and the night period was defined as the time between 22:00 and 05:59. All the participants were asked to keep regular hours based on the criteria.

### 2.4 Statistical analysis

Statistical analysis was performed using IBM SPSS 25.0. Descriptive statistical results were shown as mean ± SD for continuous data, number, and percentage for categorical data. Chi‐square tests were used for categorical data, and independent *t*‐tests or rank‐sum tests were used for continuous data, depending on whether the data met the normal distribution and homogeneity of variance. *p* < .05 indicates that the difference is statistically significant.

## 3 RESULTS

### 3.1 Patient characteristics

In the present study, a total of 37 eligible patients with ZAP were enrolled. Subjects in the control group with the same gender composition and similar age were selected from healthy people. Most patients and normal controls were female (59.5%, *N* = 37). Patients had a mean age of 70.6 years old and mean BMI of 23.8 kg/m^2^. Control group had a mean age of 70.6 years old, and mean BMI of 23.6 kg/m^2^. Some patients suffered from pain that lasted less than 3 months after the rash onset, which was defined as acute herpetic neuralgia or subacute herpetic neuralgia (*N* = 21, 56.8%); the other part of patients with pain after the rash for more than 3 months was diagnosed with PHN (*N* = 16, 43.2%). General information of sex, age, BMI, and past illnesses had no statistical difference in either group. ZAP patients had a mean VAS score of 6.57 (Table 1).

**TABLE 1:** General information of observation and control groups.

	Observation group (*N* = 37)	Control group (*N* = 37)
Gender, *n* (%)		
Male	15 (40.5)	15 (40.5)
Female	22 (59.5)	22 (59.5)
Age, mean (SD), years	70.6 ± 10.4	70.6 ± 12.1
BMI, mean (SD), kg/m^2^	23.8 ± 3.3	23.6 ± 3.4
Hypertension, *n* (%)	19 (51.4)	16 (43.2)
Hyperlipemia, *n* (%)	21 (56.8)	20 (54.1)
Duration, *n* (%)		
≤3 months	21 (56.8%)	–
>3 months	16 (43.2%)	–
VAS, mean (SD)	6.57 ± 1.12	–

Abbreviations: SD, standard deviation; VAS, visual analogue scale.

### 3.2 Evaluation of HRV

#### 3.2.1 Comparison of HRV data between two groups

Patients with ZAP had increased mean heart rate compared with control group (73.0 ± 9.5 vs. 65.9 ± 6.5, *p* < .001). As shown in Table 2, the SDNN, RMSSD, LF, and HF of the patients were significantly lower than those of control group (*p* < .05) in all periods, indicating great decrement in autonomic function, especially parasympathetic activity. The ratio of LF/HF was similar in patients and healthy people in all periods, with no statistically significant differences, indicating that growth of sympathetic activity was not observed (Table 2).

**TABLE 2:** Effects of zoster‐associated pain (ZAP) on heart rate variability (HRV).

Parameters	Observation group			Control group		
	All day	Day	Night	All day	Day	Night
Time domains						
Mean heart rate	73.0 ± 9.5^***^	–	–	65.9 ± 6.5	–	–
SDNN	115.8 ± 23.9^***^	99.2 ± 24.0^***^, ^c^	85.5 ± 25.2^***^	146.0 ± 19.0	122.5 ± 17.1	124.0 ± 17.6
RMSSD	42.3 ± 30.8^*^	42.8 ± 39.1^*^	46.4 ± 43.6^**^	49.3 ± 22.9	47.2 ± 23.3^b^	53.3 ± 26.0
Frequency domains						
LF	270.7 ± 567.6^***^	250.7 ± 509.1^***^, ^c^	308.3 ± 679.5^***^	436.2 ± 266.6	412.7 ± 234.6^c^	513.6 ± 473.3
HF	209.6 ± 605.8^***^	166.3 ± 540.8^***^, ^c^	292.2 ± 749.6^***^	228.6 ± 181.0	175.3 ± 160.1^c^	331.5 ± 272.9
LF/HF	2.2 ± 1.5	2.7 ± 1.6^c^	1.8 ± 1.9	2.4 ± 1.5	3.2 ± 2.0^c^	2.0 ± 1.6

*Note*: Absence of label indicates a nonsignificant difference between two groups or two periods (p > .05).

Abbreviations: HF, high frequency; LF, high frequency; RMSSD, root mean square of the differences in successive; SDNN, standard deviation of normal‐to‐normal.

^a^ *p* < .05 for day period versus night period.

^b^ *p* < .01 for day period versus night period.

^c^ *p* < .001 for day period versus night period.

^∗^ *p* < .05 for observation group versus control group.

^∗∗^ *p* < .01 for observation group versus control group.

^∗∗∗^ *p* < .001 for observation group vs. control group.

#### 3.2.2 Circadian rhythms of 24‐h HRV in two groups

In the control group, SDNN had no significant difference between day and night suggesting similar autonomic function in two periods (*p* > .05). The levels of RMSSD, LF, and HF during the day were lower than those at night (*p* < .05). The ratio of LF/HF in day period was significantly higher than that in night period indicating sympathetic dominance during the day (*p* < .001).

In the observation group, LF and HF in the daytime were decreased versus those in the nighttime (*p* < .05). However, unlike control group, the SDNN of ZAP patients in night period was reduced versus that in day period, indicating unbalanced autonomic function during two periods (*p* < .001). No significant difference of RMSSD between two periods was observed in patients with ZAP, indicating a loss of parasympathetic advantage in the night (*p* > .05). Patients had a higher ratio of LF/HF during the day consistent with control group (*p* < .001) (Table 2).

## 4 DISCUSSION

To our best knowledge, our study is one of the rare and comprehensive attempts to explore circadian changes of autonomic function in patients with ZAP via 24‐h HRV analysis. In the present study, we showed some significant evidence about decreased HRV in ZAP patients, which might indicate that ZAP weakens autonomic function. At the same time, this study revealed that circadian rhythms of ANS were disturbed when people suffered from ZAP.

SNSs and PNSs are two subdivisions of ANS and innervated by the cerebral cortex and hypothalamus to manage the physiological activities of organs with a relationship of confrontation and coordination (Gibbons, 2019; Wehrwein et al., 2016). Painful disorders affect autonomic function. Martinez‐Lavin et al. (1998) found that fibromyalgia patients had decreased HRV due to an increased nocturnal predominance of LF, indicating that the balance between sympathetic and parasympathetic tonus switched to the sympathetic side. A study demonstrated that, compared with healthy controls, individuals with trigeminal neuralgia had a greater increase in cardiac sympathetic activity and a greater decrease in cardiac parasympathetic activity during a cold pressor test (Léonard et al., 2015).

The underlying mechanism of interaction between pain and ANS was complex and still not clearly understood. According to available research, many pain‐related regions in brain are linked with ANS (Tracey & Mantyh, 2007). The forebrain level, anterior limbic areas, midbrain periaqueductal gray, and posterior and lateral hypothalamic areas were associated with the integration of pain modulation and autonomic control (Arslan & Unal, 2022; Cortelli et al., 2013; Pereira et al., 2010). The SNS not only regulates autonomic function but also plays an important role in the conduction of sensory pathways. After peripheral nerve damage, sympathetic nerve fibers sprout in DRG and areas of impaired nerves and tissue inflammation and form special structures with sensory neurons to contribute to the development of chronic neuropathic pain (Arslan & Unal, 2022; Xie et al., 2010).

In the comparison of HRV of whole day, day period, and night period in both groups, the present study found a decreased overall autonomic function in patients with ZAP, especially PNS function, according to SDNN, RMSSD, and HF. LF could be influenced by both PNS and SNS with various degrees, depending on activities and conditions. Therefore, the LF/HF ratio is a more reasonable metric to estimate the function of SNS and the balance between SNS and PNS. However, an increase of sympathetic activity was not observed. We have considered two reasonable explanations: Either the improvement in sympathetic function was not captured due to the small sample size, or both sympathetic and parasympathetic functions showed a decrease while the balance between the two was not disrupted. In fact, most studies suggested that painful disorders manifest an increased sympathetic activity (Léonard et al., 2015; Santos‐De‐Araújo et al., 2019). In concordance with previous studies (Adler‐Neal et al., 2020), our study showed a decrease in parasympathetic activity in ZAP patients. The vagus nerve plays an important role in both the ascending pathway of nociceptive stimuli to the center and descending inhibitory pathways within the dorsal horn of the spinal cord (Koenig, Falvay et al., 2016; Koenig, Loerbroks et al., 2016). Decreased vagus nerve activity results in more nociceptive input. This may reveal one of the pathological mechanisms of central sensitization in patients with postherpetic neuralgia.

In the observation of circadian rhythms, sympathetic activity had no increase during day and night according to LF‐to‐HF ratio. The RMSSD is used to estimate the vagally meditated changes and is strongly associated with the HF power (Koenig, Falvay et al., 2016; Nahman‐Averbuch et al., 2014). This study showed a reduced autonomic function and a loss of parasympathetic advantage in patients with ZAP during the night. We considered that the lower ANS of night was linked with the decrease of parasympathetic activity. It might be a reason why patients with neuralgia present with increased pain at night. In addition, the decline in function of PNS gives rise to aggravate ZAP accompanying symptoms, such as anxiety, depression, and insomnia, which are the main causes of psychosocial dysfunction (Adler‐Neal et al., 2020; Chaves et al., 2021; Mork et al., 2013).

Several strengths and shortcomings should be mentioned in the present study. To accurately observe the circadian rhythm of HRV, 24‐h Holter was used in this study to measure HRV parameters. Compared to short‐term HRV, subjects have sufficient time to adapt to the device to minimize the discomfort of wearing the device, which interferes with results. 24‐h Holter has a clear division of day and night, but not all participants keep regular hours in the light of this criterion. The results may be affected by this condition. Small sample size impedes the statistical power of the analyses. Perhaps augmenting of sample allows us to detect other ANS abnormalities (e.g., correlation between the degree of pain and HRV parameters).

## 5 CONCLUSION

The results of this study indicated that ZAP contributes to changes in the ANS, resulting in decreased autonomic function. The patients with ZAP lost parasympathetic advantage and had a worse ANS during the night. More interactions between ANS functions and ZAP remain to be explored.

## AUTHOR CONTRIBUTIONS

**Peng Mao**: Writing—review and editing. **Hui‐min Hu**: Investigation; writing—original draft. **Ran Li**: Data curation. **Yuan‐Jing Zhang**: Data curation. **Yi Zhang**: Data curation. **Yi‐Fan Li**: Investigation. **Bi‐fa Fan**: Methodology; Writing—review and editing.

## CONFLICT OF INTEREST STATEMENT

The authors declare that there are no conflicts of interest regarding the publication of this article.

### PEER REVIEW

The peer review history for this article is available at https://publons.com/publon/10.1002/brb3.3489.

## Data Availability

The data that support the findings of this study are available on request from the corresponding author. The data are not publicly available due to privacy or ethical restrictions.
